# Neuropsychiatric Symptoms Mimicking Dementia in a Patient Treated With Imatinib

**DOI:** 10.1002/acn3.70296

**Published:** 2025-12-29

**Authors:** Ashley Jones, Samuele Bonomi, Keith E. Stockerl‐Goldstein, Randall J. Bateman

**Affiliations:** ^1^ Saint Louis University School of Medicine St. Louis Missouri USA; ^2^ Department of Neurology Washington University School of Medicine in St. Louis St. Louis Missouri USA; ^3^ Knight Alzheimer Disease Research Center Washington University in St. Louis St. Louis Missouri USA; ^4^ Division of Oncology Washington University School of Medicine in St. Louis St. Louis Missouri USA

**Keywords:** behavioral neurology, chronic myeloid leukemia, dementia, neuropsychiatry, tyrosin kinase inhibitors

## Abstract

Tyrosine kinase inhibitors are the cornerstone of chronic myeloid leukemia treatment. Newer agents have more potency and a broader spectrum of action, but also a higher potential for neuropsychiatric side effects. We present a case of a patient on imatinib who developed progressive cognitive, mood, and behavioral alterations. He was investigated for dementia and psychiatric disorders but ultimately improved only after discontinuing imatinib. His decompensation years after therapy start, followed by complete and rapid remission, is atypical. We also review the most recent neurobiological findings on the role of tyrosine kinase alterations in dementia and their importance for mood regulation.

## 1 Case Report

We present the case of a right‐handed man who was diagnosed with Chronic Myeloid Leukemia (CML) at the age of 53. He has a strong family history of Alzheimer's disease (AD), and his past medical history is notable for depression, anemia, hypertension, and concussion. He was started on imatinib 400 mg daily, which he tolerated well without notable side effects, and his CML entered remission. His other medications included aspirin, atenolol, pitavastatin, nortriptyline, omeprazole, and fluoxetine. He worked as a computer salesman and was able to manage all his activities of daily living.

Five years later he was referred to Neurology after his wife reported concerns about increasing irritability and difficulty recalling names. His neurological exam was unremarkable except for decreased blinking and brisk patellar reflexes. Some of the neuropsychological tests were borderline normal range, such as the Mini‐Mental State Examination (27/30) and Logical Memory (8), whereas Word List Recall (3) was below normal [1]. These findings suggested subtle deficits in short‐term memory, despite an overall impression of grossly normal cognitive function and a Clinical Dementia Rating (CDR) of 0, indicating no dementia (Table 1). A brain MRI revealed no pathological findings, including near average regional brain volumes. His bloodwork showed normal levels of vitamin B12 (512 pg/mL), folate (24 ng/mL), and TSH (0.89 μIU/mL). He was recommended behavioral modifications, such as to improve sleep hygiene and exercise. Repeat neuropsychological testing 1 year later was stable, but his wife reported ongoing memory issues and irritability.

**TABLE 1:** Neurocognitive test scores since imatinib initiation with test‐specific normal ranges.

Test	Year 5	Year 6	Year 9	Year 10	Year 10^b^	Normal range
Boston naming	15	15	15	15	15	14.0–15.2
Digit symbol	40	40	^a^	33	37	35.1–55.7
Logical memory	8	12	7	9	10	8.8–17.2
Mini‐mental state exam	27	30	23/23	28	30	27–30
Short blessed test	0	0	2	0	0	0–4
Trail making Test A (s)	28	27	^a^	24	26	29.6–54.6
Trail making Test B (s)	53	56	^a^	51	50	59.4–150.6
Verbal fluency	14	14	14	17	21	13.2–22.8
Word list memory task	15	16	21	14	14	17.4–24.8
Word list recall	3	6	7	4	6	5.4–9.0

Abbreviation: s, seconds.

^a^ Test not performed (virtual visit).

^b^ Imatinib discontinued.

**FIGURE 1:**
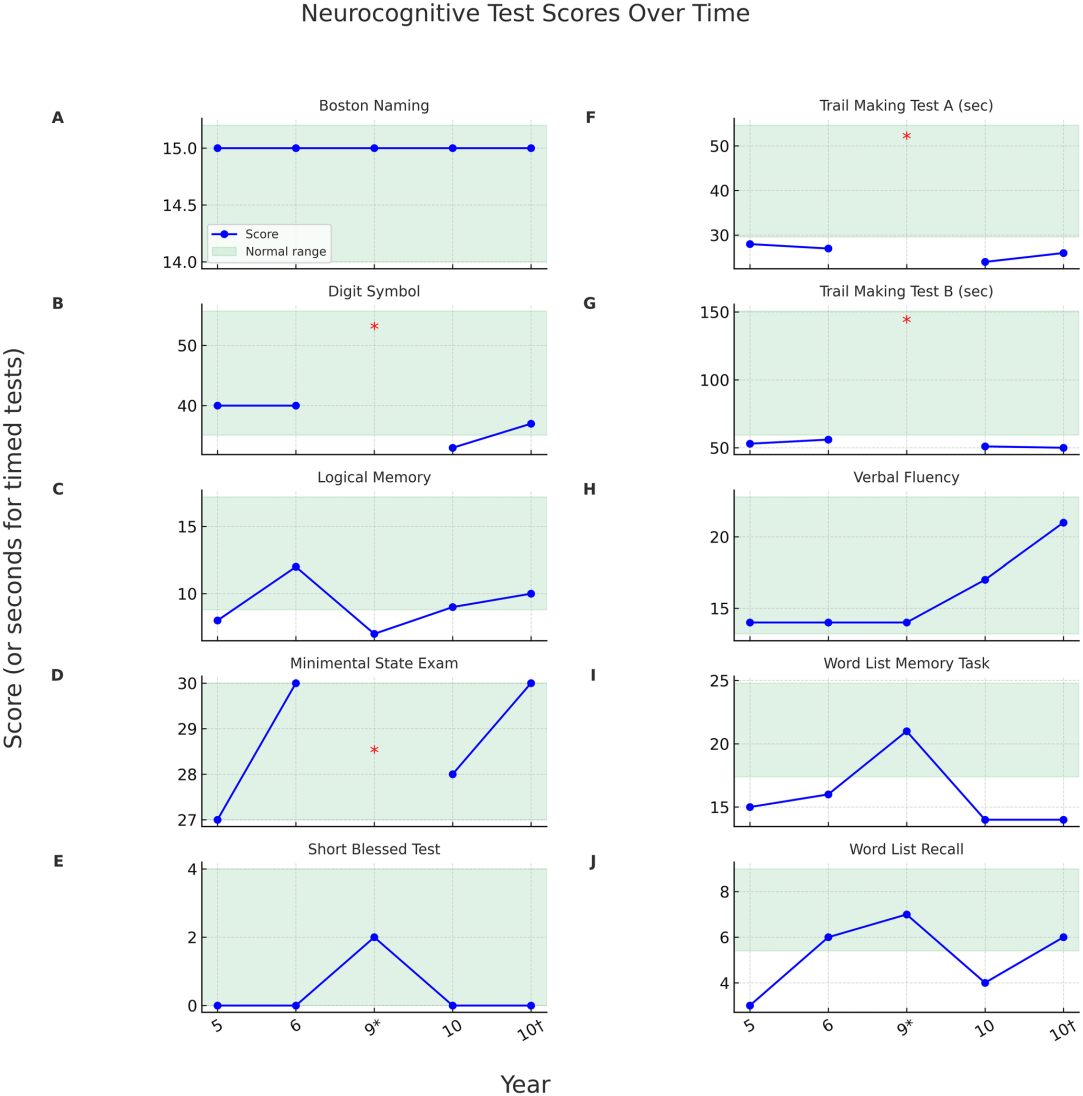
Neurocognitive test scores since imatinib initiation with test‐specific normal ranges (green shading). Higher scores indicate better performance, except for Trail Making Tests A and B, where lower times indicate better performance. *Virtual visit (testing limited); †Off imatinib.

After 3 years, the patient was re‐referred to a dementia specialty clinic after a severe decline in his mood, personality, and cognitive function over the previous months. He was no longer able to use a phone or computer, struggled to retain new information, had bothersome insomnia, and exhibited persistent irritability as well as intermittent aggression towards close family members. Lurasidone had been started by a psychiatrist but proved ineffective. Repeat neuropsychological testing was stable from the past, which was inconsistent with a progressive neurodegenerative dementia syndrome (Figure 1).

Given no improvement with behavioral modifications and psychotherapy, the dementia specialist raised the potential that imatinib could be contributing to the patient's neuropsychiatric symptoms. In coordination with Hematology/Oncology, a shared decision was made to discontinue imatinib after 11 years of continuous use. Within 2 months of discontinuation, significant improvement in mood and cognition was observed. The patient was significantly less irritable and aggressive, started helping with tasks in the home, and resumed using technology. 3 months later, his mood and cognition had returned to his normal baseline. In the setting of improved function and return to normal personality after discontinuation of the drug, iatrogenic encephalopathy secondary to imatinib was formally diagnosed. 4 years later, he continued to retain full independence and his family reported no cognitive or behavioral concerns. Close clinical monitoring and laboratory testing confirmed continued CML remission.

## 2 Discussion

Tyrosine kinase inhibitor (TKI) therapy is the frontline treatment for CML. This class of medication works by inhibiting the aberrant BCR‐ABL1 fusion gene, the main genetic mutation responsible for the pathologic myeloproliferation that characterizes CML [2]. The six main TKIs used in the treatment of CML include imatinib, dasatinib, nilotinib, bosutinib, ponatinib, and asciminib. These drugs span four generations of TKI therapy, with imatinib being the first agent to be approved, and asciminib being the only fourth generation agent available. The 10‐year mortality rate has dropped from 10%–20% to 1%–2% with TKI therapy [3], transitioning CML from a highly fatal cancer to a chronically manageable disease in most patients. Newer agents tend to have higher potency, broader spectrum of kinase inhibition, and more coverage against resistance mutations that cancer cells may develop. Common side effects include cytopenias, fatigue, muscle cramps, edema, skin changes, and GI tract symptoms [4].

With increasing use of TKIs for CML, reports started to emerge regarding the potential for causing neuropsychiatric symptoms. Early reports include a case series of individuals who had experienced depression, anxiety, and insomnia during months‐long treatment with dasatinib or imatinib, which resolved after discontinuing or reducing the TKI dosage and resurfaced following TKI‐rechallenge [5]. A subsequent study assessed that 19 out of 99 patients treated with dasatinib at standard dose experienced memory changes after a median of 41 months [6]. Agitation has also been reported as a potential side effect of dasatinib [7].

From a neurobiological standpoint, it appeared that TKIs, such as dasatinib, that target a broader spectrum of kinases and have high penetration of the blood–brain barrier, also have a higher risk of causing neuropsychiatric symptoms via dysregulation of several cellular pathways [8]. Specifically, the Abl kinase that all TKIs target is involved in the regulation of neuronal activity, with mechanisms that include tau phosphorylation [9], parkin phosphorylation [10], and cell death regulation [11]. As compared to the first generation imatinib, dasatinib can also target the Src family of kinases, which may be involved in AD. It has been demonstrated in vitro that increased tyrosine phosphorylation of tau and microtubule‐associated protein 2c (MAP2c) happens in response to Aβ, and that inhibition of the Src family of kinases blocks the tyrosine phosphorylation [12]. Later, Src was linked to synaptic plasticity [13] and microglial activity [14]. More recently, a multi‐center effort showed that the T isoform of the Fyn tyrosine kinase, a member of the Src family, is upregulated in the cortex of patients with AD, Parkinson's disease dementia, and dementia with Lewy bodies [15]. This selective increase in FynT expression correlated with hallmark pathological features such as phosphorylated tau and soluble β‐amyloid, as well as markers of neuroinflammation including microglia and astrocyte activation. Supported by this evidence, tyrosine kinases have started to receive attention as possible therapeutic targets, with a recent study showing that c‐Abl tyrosine kinase inhibition leads to a reduction in amyloid plaque formation and astrogliosis in a mouse model of AD [16]. Although selective tyrosine kinase inhibition appears to have therapeutic potential in the presence of AD pathology in which kinase expression is dysregulated, we agree with other authors that the effect of this inhibition in a healthy brain and in a less specific fashion may be the reason behind the neuropsychiatric adverse effects of TKI drugs [8]. In line with this concept, a recent review summarized the role of the interplay between tyrosine kinase B isoforms and brain‐derived neurotrophic factor (BDNF) signaling, which could contribute to mood dysregulation and cognitive impairment [17].

Neuropsychiatric symptoms as a side effect of TKIs may have a highly variable timeline, with some occurring in as little as 2 months of therapy and potential resolution in less than a week of discontinuation [7, 18], although resolution is often not complete and especially mood changes can persist after discontinuing the therapy. A more recent study assessed the differences in the side effect profile of dasatinib, nilotinib, and ponatinib [19]. The authors showed that neuropsychiatric changes are more prevalent and significant in the 6 to 18 months window after TKI therapy initiation, but may appear throughout the therapy course. The newer agent ponatinib had worse scores for patient‐reported insomnia, sadness, and memory issues.

In the case we presented, it is relevant to note the chronic development of mood, cognitive, and behavioral changes, which in a patient with personal history of depression and significant family history for dementia initially led to consider his symptoms as possibly related to excess worry about his health, a mood disorder, or a slowly progressive primary dementia for several years. It also highlights the potential for decompensation of these manifestations even a long time after therapy start. Different tools exist to assess the likelihood of a medication causing an adverse effect, such as the Naranjo Probability Scale [20], which includes 10 questions that are scored between −1 and +2 depending on a yes or no answer, which can classify the association as definite, probable, possible, or doubtful. Based on this scale, it is probable that imatinib caused the neuropsychiatric changes in the case we present. Lastly, the rapidity and the completeness of the improvement of this patient's symptoms, despite having been on therapy for many years, are noteworthy compared to other patients in the literature. Another group recently reported resolution of significant neuropsychiatric changes in a patient after holding dasatinib, with no relapsing symptoms after starting bosunitib [8], highlighting the existence of a reasonable alternative strategy by trying a different TKI if the initially chosen agent leads to intolerable side effects. Careful monitoring of CML remission after holding TKI therapy is also a reasonable alternative, as was done in this case.

In conclusion, providers taking care of patients who are being treated with TKIs need to be aware of the possible neuropsychiatric manifestations, especially cognitive, mood, and behavioral changes, including severe irritability, insomnia, and cognitive impairment that patients may experience even years after therapy starts. In a landscape in which testing for biomarkers of dementia is becoming more widely available [21, 22], but still potentially less accessible depending on the geographic area [23], our report aims to raise awareness of the possible neuropsychiatric adverse effects of TKIs to help prevent unnecessary testing or misattribution to psychiatric or neurodegenerative disorders.

## Author Contributions

A.J., S.B., K.E.S.‐G., and R.J.B. have all contributed to the design, data collection, drafting, and editing of this study.

## Funding

This work was supported by resources and effort provided by the Tracy Family SILQ Center established by the Tracy Family, Richard Frimel & Gary Werths, GHR Foundation, Pat and Jane Tracy, Anonymous, Anne & Ray Capestrain, Community Foundation Serving West Central Illinois and Northeast Missouri, JTL Family Fund, Payne Family, Mary & Jay Sullivan, Tracy Family Foundation, Catherine & Tom Tracy, Community Foundation for the Land of Lincoln, Jim & Jil Tracy, Joe & Jill Tracy, Sonja & Robert M. Willman, Boniface Foundation, Jean & Michael Buckley, Ann Liberman, Clemence S. Lieber Foundation, Mary Schoolman & Dr. James Hinrichs, and Susan & Scott Stamerjohn brought together by The Foundation for Barnes‐Jewish Hospital.

## Consent

The patient provided written consent for the publication of this manuscript and had the chance to see the submitted version. The patient has been informed that if published, the report will be freely accessible to anyone 1 year after publication and thus confidentiality cannot be guaranteed despite all efforts to disguise personal details. The authors confirm that details of the case presented, including dates and locations, have been disguised to protect patient privacy.

## Conflicts of Interest

Dr. Keith E. Stockerl‐Goldstein has received research funding from Glycomimetics, Janssen Pharmaceuticals, and Nexcella Inc. Dr. Randall J. Bateman has received research funding from Avid Radiopharmaceuticals, Janssen, Roche/Genentech, Eli Lilly, Eisai, Biogen, AbbVie, Bristol Myers Squibb, and Novartis. Washington University and R.J.B. have equity ownership interest in C2N Diagnostics and receive income based on technology licensed by Washington University to C2N Diagnostics. R.J.B. receives income from C2N Diagnostics for serving on the scientific advisory board. R.J.B. serves as an unpaid member on scientific advisory boards for Roche and Biogen.

## Data Availability

The data that support the findings of this study are available on request from the corresponding author. The data are not publicly available due to privacy or ethical restrictions.
